# Gene-Specific Assessment of Guanine Oxidation as an Epigenetic Modulator for Cardiac Specification of Mouse Embryonic Stem Cells

**DOI:** 10.1371/journal.pone.0155792

**Published:** 2016-06-01

**Authors:** Joonghoon Park, Jong Woo Park, Hawmok Oh, Fernanda S. Maria, Jaeku Kang, Xiuchun Tian

**Affiliations:** 1 Center for Regenerative Biology and Department of Animal Science, University of Connecticut, Storrs, Connecticut, 06269, United States of America; 2 Research Center for Epigenome Regulation, School of Pharmacy, Sungkyunkwan University, Suwon, 440746, Republic of Korea; 3 Department of Animal Reproduction, College of Veterinary Medicine, University of Sao Paulo, Sao Paulo, 05508, Brazil; 4 Department of Pharmacology, College of Medicine, Konyang University, Daejeon, 302718, Republic of Korea; Guangzhou Institute of Biomedicine and Health, CHINA

## Abstract

Epigenetics have essential roles in development and human diseases. Compared to the complex histone modifications, epigenetic changes on mammalian DNA are as simple as methylation on cytosine. Guanine, however, can be oxidized as an epigenetic change which can undergo base-pair transversion, causing a genetic difference. Accumulating evidence indicates that reactive oxygen species (ROS) are important signaling molecules for embryonic stem cell (ESC) differentiation, possibly through transient changes on genomic DNA such as 7,8-dihydro-8-oxoguanine (8-oxoG). Technical limitations on detecting such DNA modifications, however, restrict the investigation of the role of 8-oxoG in ESC differentiation. Here, we developed a Hoogsteen base pairing-mediated PCR-sequencing assay to detect 8-oxoG lesions that can subsequently cause G to T transversions during PCR. We then used this assay to assess the epigenetic and transient 8-oxoG formation in the *Tbx5* gene of R1 mouse ESCs subjected to oxidative stress by removing 2-mercaptoethanol (2ME) from the culture media. To our surprise, significantly higher numbers of 8-oxoG-mediated G∙C to C∙G transversion, not G∙C to T∙A, were detected at 7^th^ and 9^th^ base position from the transcription start site of exon 1 of *Tbx5* in ESCs in the (-)2ME than (+)2ME group (*p* < 0.05). This was consistent with the decrease in the amount of amplifiable of DNA harboring the 8-oxoG lesions at the *Tbx5* promoter region in the oxidative stressed ESCs. The ESCs responded to oxidative stress, possibly through the epigenetic effects of guanine oxidation with decreased proliferation (*p* < 0.05) and increased formation of beating embryoid bodies (EBs; *p* < 0.001). Additionally, the epigenetic changes of guanine induced up-regulation of *Ogg1* and *PolB*, two base excision repairing genes for 8-oxoG, in ESCs treated with (-)2ME (*p* < 0.01). Together, we developed a gene-specific and direct quantification assay for guanine oxidation. Using oxidative stressed mouse ESCs, we validated this assay and assessed the epigenetic effects of 8-oxoG by studying expression of DNA repair genes, ESC proliferation, and EB formation.

## Introduction

Mammals are composed of numerous cell types with distinct patterns of gene expression. Such expression patterns are transmittable from parental to daughter cells through epigenetic memories [1, 2]. Epigenetic modifications are essential in development [3]; however, they have also emerged as causes for human diseases including cancers, developmental disorders, and autoimmune ailments [4, 5]. The molecular basis of epigenetics involves DNA and histone modifications [6]. More than 60 histone alterations have been reported so far including acetylation, methylation, phosphorylation and ubiquitination on different lysine residues [7]. Compared to these complexities, epigenetic modification on mammalian DNA is limited to methylation on the C5 position of cytosines adjacent to guanines (CpG). Modifications on other bases and their roles remain to be investigated in mammals [8].

Reactive Oxygen Species (ROS) are produced from aerobic metabolism in the mitochondria and can also be induced by exogenous agents such as ionizing radiation or oxidizing chemicals. In mitochondria, single electron reduction of oxygen leads to the formation of ROS such as superoxide ions, hydrogen peroxide and highly reactive hydroxyl radicals [9, 10]. Appropriate levels of ROS in cells act as signaling molecules for various cellular events [11]. Excessive ROS, however, causes oxidative stress by damaging macromolecules such as proteins, lipids and DNA and is linked to various pathological conditions, including aging, carcinogenesis, neurodegenerative and cardiovascular diseases [12]. The most common oxidative modification on DNA is the generation of 7, 8-dihydro-8-oxoguanine (8-oxoG), which can be formed when guanine reacts with hydroxyl radicals and/or hydrogen peroxide. Among C4, C5, and C8 positions of guanine, C8 is the most favored position for hydroxyl radical adduct, resulting in 8-oxoG [13]. In general, 8-oxoG is regarded as a DNA lesion and can be recognized and removed by 8-oxoguanine DNA glycosylase (*Ogg1*) through base excision repair [14]. It has been known that 8-oxoG itself is nontoxic and weakly mutagenic [15]. However, 8-oxoG could be transcriptional mutagenic when incorporation of incorrect nucleotides occurs during transcription, leading to the generation of mutant proteins [16] such as the *Ras* oncogene. Additionally, 8-oxoG was reported to affect the expression of estrogen-induced genes and/or hypoxia-inducible genes by chromatin relaxation [17, 18]. Furthermore, 8-oxoG produced by lysine de-methylating enzyme drives the expression of *Myc* target genes [19, 20]. These observations imply the transcriptional regulation and/or epigenetic properties of 8-oxoG. If not repaired, however, the epigenetic 8-oxoG can also cause genetic changes. For example, during DNA replication 8-oxoG can induce the formation of intermediate triple-stranded DNA through Hoogsteen base pairing [21], which is a transient variation of the Watson-Crick base pairing scheme. This occurs when the N7 position of the purine base acts as a hydrogen bond acceptor and C6 amino group as a donor, resulting in syn to anti conformation between nucleosides [22]. As a result, 8-oxoG has higher affinity to adenine than cytosine, leading to G∙C to T∙A transversion in daughter cells [23, 24].

Several methods are currently available to measure 8-oxoG in genomes. These include HPLC equipped with electrochemical detection (HPLC-ECD), ligation-mediated PCR (LM-PCR), mass spectrometry, and DNA immunoprecipitation (IP) [25]. However, most of them are limited to analyze large fragments of DNA ranging from kilo-bases to mega-bases; therefore, it is difficult to study base-specific oxidative modifications on DNA. The recently introduced single-molecule, real-time (SMRT) DNA sequencing technique provides quantitative measures of DNA modification by analyzing the interpulse duration of DNA polymerase when it replicates damaged DNA bases including 8-oxoG [26]. This method, however, is still an indirect detection of the oxidative DNA lesions. Here, we aimed to use the Hoogsteen base-pairing property of 8-oxoG to develop a gene-specific assay for 8-oxoG at the single base level.

Embryonic stem cells are capable of self-renewal and differentiation into cell types of the three germ layers and thus hold enormous potential for therapeutic applications of cell-based regenerative medicine. Upon differentiation, the number of mitochondria in ESCs increase with concomitant increase in ATP and ROS [27]. ROS has been shown to signal ESC differentiation, especially into cardiomyocytes [28, 29]. Therefore, differentiating ESCs are a suitable model to assess the epigenetic effect of 8-oxoG induced by oxidative stress.

In this study, we evaluated 8-oxoG as an alternative epigenetic modification on DNA. To this end, we first established a Hoogsteen base pairing-mediated PCR-sequencing assay to detect 8-oxoG modifications at the single base level. Subsequently, we introduced oxidative stress on R1 mouse ESCs and measured the levels of 8-oxoG in the 5’-proximal regions of 4 genes, *Tbx5*, *Mef2C*, *Nkx2-5*, and *Gata4*, all well-known biomarkers of cardiac specification. Chromatin immunoprecipitation accompanied with quantitative PCR (ChIP-qPCR) with anti-human Ogg1 antibody against *Tbx5* promoter region was also conducted. Lastly we studied the effects of oxidative stress and 8-oxoG on ESC differentiation into cardiomyocytes and on the levels of mRNA and protein for DNA repair genes, *Ogg1* and *PolB*.

## Materials and Methods

### Establishment of Hoogsteen base pairing-mediated PCR-sequencing assay

pUC19oxoG(+), a plasmid derived from pUC19 containing a single 8-oxoG lesion in the Hind III site, was prepared by using Phusion site-directed mutagenesis kit (Finnzymes, Keilaranta, Finland). Briefly, an 8-oxoG lesion in the Hind III site (AAGCTT) was introduced into a control plasmid (pUC19_lacαL8Stop) by G to 8-oxoG substitution in the PCR primers. Such substitution renders the loss of the Hind III restriction site while the control plasmid maintains it. The primers used were as follows: 5’-CTGCAGGCATGTAAoxoGCTTGGCGTA-3’. Control primers did not contain such substitution and were used to generate pUC19oxoG(-) to verify the site-directed mutagenesis. Primers were phosphorylated at 5’-end and purified by PAGE (Sigma-Genosys, Woodlands, TX). PCR-based mutagenesis was carried out with 1.6 ng of control plasmid in a 50 μL reaction mix according to manufacturer’s instructions as follows: initial denaturation was performed at 98°C for 30 sec, followed by 40 cycles of denaturation at 98°C for 10 sec, annealing/extension at 72°C for 45 min, and final extension at 72°C for 5 min. PCR products were ligated by using 800 U of T4 DNA ligase (New England Biolab, Ipswich, MA) at 25°C for 2 hr to generate circular pUC19oxoG(+). To validate the 8-oxoG lesion, a 300 bp region harboring the Hind III site was amplified from pUC19oxoG(+) with the following primers: (forward) 5’-CTGCGCAACTGTTGGGAAG-3’, (reverse) 5’-GGCACCCCAGGCTTTACACT-3’. PCR was performed by using platinum *Taq* PCRx DNA polymerase (Invitrogen, Carlsbad, CA) with initial denaturation at 94°C for 3 min followed by 45 cycles of denaturation at 94°C for 30 sec, annealing at 57°C for 30 sec, extension at 72°C for 30 sec, and final extension at 72°C for 5 min. PCR products were subjected to Hind III digestion (20U at 37°C for 2 hr) and sequencing by using the BigDye Terminator v1.1 Cycle Sequencing kit with ABI PRISM Model 3700 (Applied Biosystems Inc., Foster City, CA).

### Induction of oxidative stress in R1 mouse ESCs

R1 mouse ESCs were purchased from the American Type Culture Collection (ATCC). It was initially established from a 3.5 day blastocyst produced by crossing two 129 substrains (129S1/SvlmJ x 129X1/SvJ). R1 mouse ESCs were cultured on mitotically inactivated CD1 mouse embryonic fibroblast (MEF) feeder layer for five passages in physiological glucose ES medium which is DMEM (Invitrogen) supplemented with 1,000 mg/L D-glucose, 15% FBS (Thermo Fisher Scientific, Waltham, MA), 100 U/mL Penicillin and 100 μg/mL Streptomycin (Invitrogen), 1000 U/mL Leukemia Inhibitory Factor (LIF, Millipore, Billerica, MA), 1% (v/v) non-essential amino acid (Millipore) and 1% (v/v) 2-mercaptoethanol (2ME, Millipore). To avoid oxidative stress in conventional ESC culture in which high level of glucose (4,500 mg/L) is present, we used the physiological level of glucose (1,000 mg/L) in our study. The adapted ESCs were seeded at 1.5 x 10^5^ cells/well in 6-well plates (Becton Dickinson Labware, Franklin Lakes, NJ), and pre-incubated in physiological glucose for 24 hrs. Oxidative stress was induced by removing 2ME from the medium. After two days of cultivation with daily medium replacement, proliferation of ESCs in the physiological glucose medium with and without 2ME was compared by cell counting after trypan blue staining. The experiment was conducted three times independently.

### 8-oxoG repair induced by oxidative stress

The 8-oxoG induced by oxidative stress can be detected by the cell’s repair mechanism which involves 8-oxoguanine glycosidase (*Ogg1*) and DNA polymerase beta (*PolB*). The expression levels of these two genes, therefore, represent an indirect measurement of the degree of guanine lesion and were determined by quantitative reverse transcription real time quantitative RT-PCR (qPCR). After oxidative stress induction, total RNA was extracted from ESCs using TRIzol (Invitrogen) and treated with DNase I (Invitrogen) to remove any possible genomic DNA contamination. RNA was purified with RNeasy mini kit (Qiagen, Valencia, CA), and cDNA was generated with Superscript III reverse transcriptase (Invitrogen). Expression levels of *Ogg1* and *PolB* were measured with the ABI 7500 Fast Real-Time PCR System (Applied Biosystems, Foster City, CA). Glyceraldehyde 3-phosphate dehydrogenase (*Gapdh*) was used as an internal control. Primer sets for qPCRs were as follows: *Ogg1* with forward primer 5’-GCTTCCCAAACCTCCATGC-3’, reverse primer 5’-TTTGGCACTGGCACGTACAT-3’, and *PolB* with forward primer 5’-AGAACGTGAGCCAGGCGA T-3’, reverse primer 5’-TCTTAGCTTCCGCTCCGCT-3’. Primers for *Gapdh* were from a previous study [30]. Five ng of cDNA in a 20 μL reaction mixture [1X SYBR Green PCR master mix (Applied Biosystems), 0.3 μM of each primer] was subjected to an initial denaturation at 95°C for 10 min, followed by 40 cycles of denaturation at 95°C for 15 sec and annealing/extension at 60°C for 1 min. Data analysis was performed with ABI prism SDS software (Applied Biosystems), and the relative expression levels of the target genes to *Gapdh* were calculated by using the comparative C_T_ method according to the manufacturer’s instructions. Quantitative measurements of *Ogg1* and *PolB* expression were performed three times independently.

The protein expression of *Ogg1* and *PolB* was tested by Western blotting. After the induction of oxidative stress, cellular proteins were extracted with PRO-PREP Protein Extraction Kit (iNtRON Biotechnology, Korea) according to the manufacturer’s instructions. Twenty μg of the extracted protein was loaded to 12% acrylamide gels. Anti-Ogg1, anti-PolB, and anti-α-tubulin primary antibodies were purchased from Abcam (Cambridge, USA), LifeSpan BioSciences (Seattle, USA), and Cell Signaling Technologies (Danvers, USA), respectively. Antibodies were diluted 1:1000 and incubated overnight. Horseradish peroxidase (HRP)-conjugated goat anti-rabbit IgG antibody (Millipore) was used as a secondary antibody.

### Levels of 8-oxoG in oxidative-stressed ESCs measured by the Hoogsteen base pairing-mediated PCR-sequencing assay

Total DNA was isolated from the oxidative-stressed ESCs using back extraction (4 M guanidine thiocyanate, 50 mM sodium citrate, 1M Tris-base) from the inter- and organic phases of RNA extraction, followed by RNase A (Invitrogen) treatment to remove any possible contamination of RNA. Isolated DNA was stored at -20°C until use. The guanine-rich regions surrounding the transcription start sites of the T box 5 gene (*Tbx5*), myocyte-specific enhancer factor 2C (*Mef2C*), NK2 homeobox 5 (*Nkx2-5*), and Gata-binding protein 4 (*Gata4*) were amplified for quantitative analysis of 8-oxoG lesions. PCR primers were designed using PSQ array design software (Biotage, Kungsgatan, Sweden), and summarized in Table 1. PCR was performed by using platinum Taq PCRx DNA polymerase (Invitrogen) with initial denaturation at 94°C for 5 min followed by 45 cycles of denaturation at 94°C for 30 sec, annealing at 60°C for 20 sec, extension at 72°C for 30 sec, and final extension at 72°C for 10 min. PCR products were subcloned into pCR2.1-TOPO plasmid, followed by transfection into TOP10F competent cells by using TOPO TA cloning kit (Invitrogen) according to the manufacturer’s instructions. After overnight incubation, white colonies were selected to verify PCR product insertion by PCR with M13 primers. Sixteen positive colonies per treatment group were used for sequencing analysis using the BigDye Terminator v1.1 Cycle Sequencing kit (Applied Biosystems). Briefly, 30 μM of purified PCR products in a 20 μL reaction mixture (1X Terminator Ready Reaction Mix, 1X BigDye Sequencing Buffer, and 0.75 μM *Tbx5* reverse primer) were subjected to an initial denaturation at 94°C for 5 min, followed by 25 cycles of denaturation at 96°C for 10 sec, annealing at 50°C for 15 sec, and extension at 60°C for 4 min. The reactant was purified with DyeEx 2.0 (Qiagen), and electrophoresis was carried out at Biotechnology/Bioservice Center at the University of Connecticut. Sequence alignment was performed using Sequencher (Gene Codes, Ann Arbor, MI) by comparing to the reference sequence (i.e., ENSMUSG00000018263 for *Tbx5*, ENSMUSG00000005583 for *Mef2C*, ENSMUSG00000015579 for *Nkx2-5*, and ENSMUSG00000021944 for *Gata4*) taken from Ensembl (www.ensembl.org). Transcription start site was predicted using the Neural Network Promoter Prediction from the Berkeley Drosophila Genome Project [31].

**Table 1:** Primer sequences for biomarker genes of cardiac specification.

Gene	Type	Sequence	Tm	Amplicon (bp)	Ensemblaccession number
*Tbx5*	Forward Primer	5’-TGCAACCACTGGCTCAGA-3’	58.8	488	ENSMUSG00000018263
	Reverse Primer	5’-TCAGCTGGGGTGCTCACA-3’	60.9		
*Mef2C*	Forward Primer	5’-CTCACCGCTTGACGATCA-3’	68.5	184	ENSMUSG00000005583
	Reverse Primer	5’-AAGTTGTTCCCGTCAGCACC-3	71.4		
*Nkx2-5*	Forward Primer	5’-GCAGGCTACTCTTAGGCAGACTT-3’	69.4	131	ENSMUSG00000015579
	Reverse Primer	5’-TTTGCACTGTGTCCTGTATCACT-3	68.6		
*Gata4*	Forward Primer	5’-AACCTCCTGGATGTGGATTTG-3’	70.0	230	ENSMUSG00000021944
	Reverse Primer	5’-CGGCTTCCCTCTCAGAATTAGATA-3’	70.9		

### Levels of 8-oxoG in oxidative-stressed ESCs measured by the ChIP-qPCR

R1 mouse ESCs (1.5 X 10^6^) were plated in 10 cm plates and maintained in the normal or oxidative stress condition as described earlier. MEFs were removed after trypsinization, R1 mouse ESCs were fixed by cross-linking using 1% formaldehyde in PBS at room temperature for 10 min and then quenched in 125 mM glycine for 5 min. The cells were washed with cold PBS and collected by centrifugation. The cell pellet was then sonicated in lysis buffer (50 mM Tris, pH 7.9, 10 mM EDTA, 1% SDS, protease-inhibitor cocktail and 1 mM DTT) to generate chromatin fragments of ~500 bp in length. The lysate was clarified by a 10-min centrifugation at 14,000 rpm and 4°C, and 20 μL of supernatant was used as input for quantitation. The remaining supernatant was diluted ten-fold in dilution buffer (20 mM Tris, pH 7.9, 2 mM EDTA, 150 mM NaCl, 0.5% Triton X-100, protease-inhibitor cocktail and 1 mM DTT) and precleared with 20 μL protein A–agarose beads at 4°C for 3 hr. The supernatant was used in immunoprecipitation at 4°C overnight with antibodies against histone H3 (Abcam) or Ogg1 (Novus, Littleton, USA). Normal IgG controls were included as negative controls. The immunoprecipitated DNA was cleared by protein digestion with 0.4 mg/mL of glycogen and 0.45 mg/mL of proteinase K (Roche, Indianapolis, USA) in Txn stop buffer at 37°C for 1 hr. The DNA was then extracted with phenol/chloroform/isoamyl alcohol (25:24:1) and ethanol precipitated. qPCR with SYBR Green (Invitrogen) was used to determine the enrichment of immunoprecipitated material relative to input with *Tbx5* promoter with (forward) 5’-TGGCTCAGAGCAGTCAACA-3’, (reverse) 5’-GATATACCCCAAACTCTTGAACCA-3’ (melting temperature = 60.2°C).

### Random differentiation of the oxidative-stressed ESCs into the cardiac lineage

At the end of the oxidative stress induction, a subset of ESCs were dissociated using 0.05% trypsin (Invitrogen) and seeded at 500 cells/microwell in the AggreWell plate (StemCell Technologies, Vancouver, Canada) for embryoid body (EB) formation in ES medium without LIF. Twenty-four hours after ESC seeding, EBs were harvested and plated onto 100-mm Petri dish (Thermo Fisher Scientific) for another 5 days of differentiation with daily medium replacement. Subsequently, fully-grown EBs were plated onto 6-well plates (Becton Dickinson) and randomly differentiated for another 7 days. During differentiation, outgrown EBs with spontaneous beating activity were counted to assess cardiac-lineage specification. The experiments were performed three times independently.

### Statistical analysis

Parametric data were tested for equal variance by F-test, and when it was not significant, data were subject to one-sided t-test (*p* < 0.05).

## Results

### Establishment of PCR-sequencing assay for gene-specific 8-oxoG lesions

**Fig 1:**
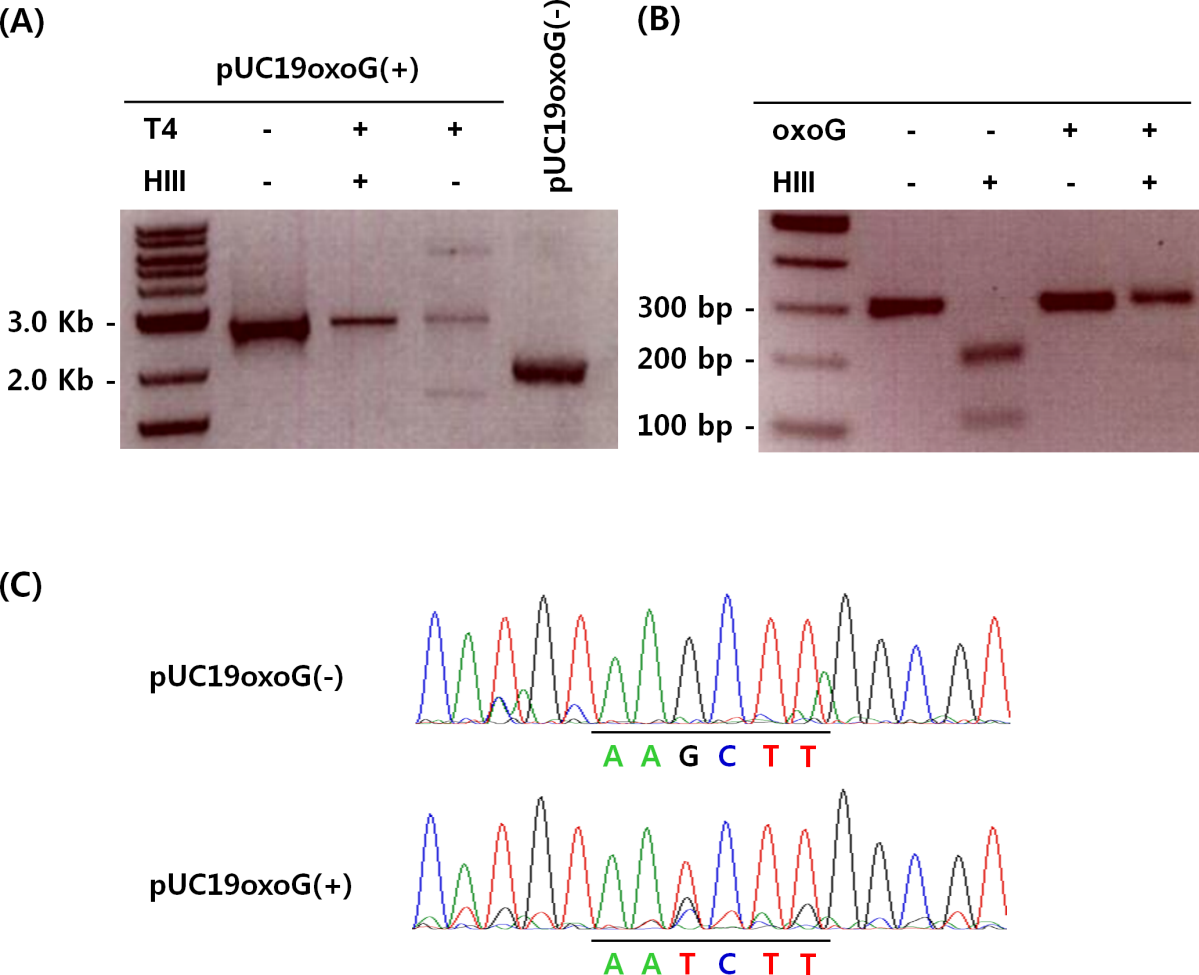
Establishment of the Hoogsteen base pairing-mediated PCR-Sequencing assay for 8-oxoG lesions in DNA. (A) pUC19oxoG(+) was prepared by site-directed mutagenesis, (B) 8-oxoG lesion was identified by Hind III resistance, and (C) Hoogsteen base pairing-mediated 8-oxoG to T transversion. T4: T4 DNA ligase, HIII: Hind III restriction enzyme.

The feasibility of Hoogsteen base pairing-mediated PCR-sequencing assay for analyzing gene-specific 8-oxoG was evaluated with a synthesized plasmid containing a single 8-oxoG lesion. As depicted in Fig 1A, 2.7 Kb linear pUC19oxoG(+) was generated by site-directed mutagenesis and whole plasmid amplification. T4 ligase produced additional bands of circular pUC19oxoG(+) probably due to structural change such as supercoiling and/or insufficient ligation. After Hind III digestion, circular pUC19oxoG(+) returned to the linear form, which implied that the pUC19oxoG(+) acquired a Hind III site that did not exist in the pUC19_LacαL8Stop control plasmid. Circular pUC19oxoG(-) was detected at about 2.0 Kb in size similar to the T4-ligated pUC19oxoG(+). Three hundred bp PCR products from these two plasmids were subjected to Hind III digestion. Enzymatic restriction generated products of 100 and 200bp from the amplicons of pUC19oxoG(-). In contrast, the PCR amplicons of pUC19oxoG(+) was resistant to Hind III digestion (Fig 1B). DNA sequencing revealed that the pUC19oxoG(-) amplicon contained a Hind III site; however, it was changed from AAGCTT to AATCTT in the pUC19oxoG(+) amplicon (Fig 1C). Therefore, these results demonstrated that 8-oxoG lesions resulted from Hoogsteen base pairing-mediated mutagenesis could be detected in amplified DNA through PCR-sequencing assay.

### Oxidative stress on R1 mouse ESC proliferation and differentiation

**Fig 2:**
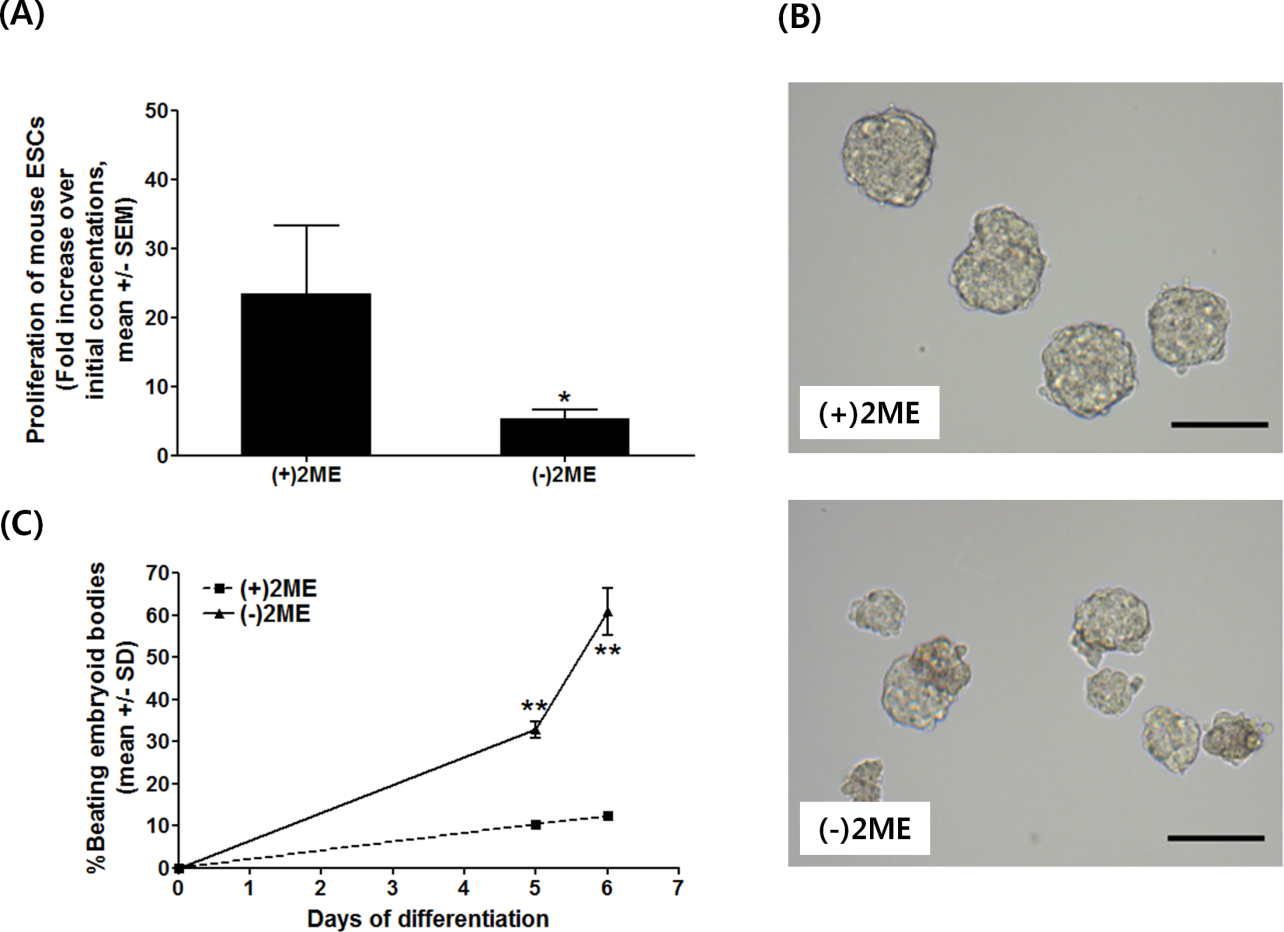
Effects of oxidative stress on R1 mouse ESC proliferation and differentiation. (A) Proliferation of ESCs after oxidative stress induced for two days by removing 2-mercaptoethanol (2ME) from ESC medium. *p < 0.05, Welch t-test. Means of fold changes with standard errors were presented. (B) Different EB morphologies from (+)2ME and (-)2ME media (bar = 100 μm) and (C) the percentages of beating EBs were observed from oxidative-stressed ESCs vs. controls (**p < 0.01, one-sided t-test).

Oxidative stress on proliferation and differentiation of ESCs was induced by removing 2ME from the ESC medium. After two days of culture, the numbers of ESCs in the (+)2ME medium increased by 24-fold over the initial cell concentration. The numbers of ESCs in the (-)2ME medium, however, increased by only 5-fold, significantly lower than that of the (+)2ME treatment (Fig 2A; *p* < 0.05). Additionally, ESCs cultivated in the (+)2ME medium aggregated and generated uniform EBs of ~100 μm in diameter; while EBs derived in the (-)2ME medium were irregular in shape and had poor morphology (Fig 2B). After 5 days of random differentiation, beating EBs appeared in both groups. The incidence of beating EBs from the (-)2ME group was significantly higher than that of the (+)2ME group (*p* < 0.001), and it reached to 60.7% over total EBs after 6 days of differentiation while in the (+)2ME group it was only 12.3% (Fig 2C). Together, these results suggest that 2ME removal from ESC culture affects the redox state of ESCs, resulting in poor proliferation and enhanced cardiomyocyte differentiation.

### Oxidative stress-induced G∙C to C∙G transversion in the *Tbx5* gene of R1 mouse ESCs

**Fig 3:**
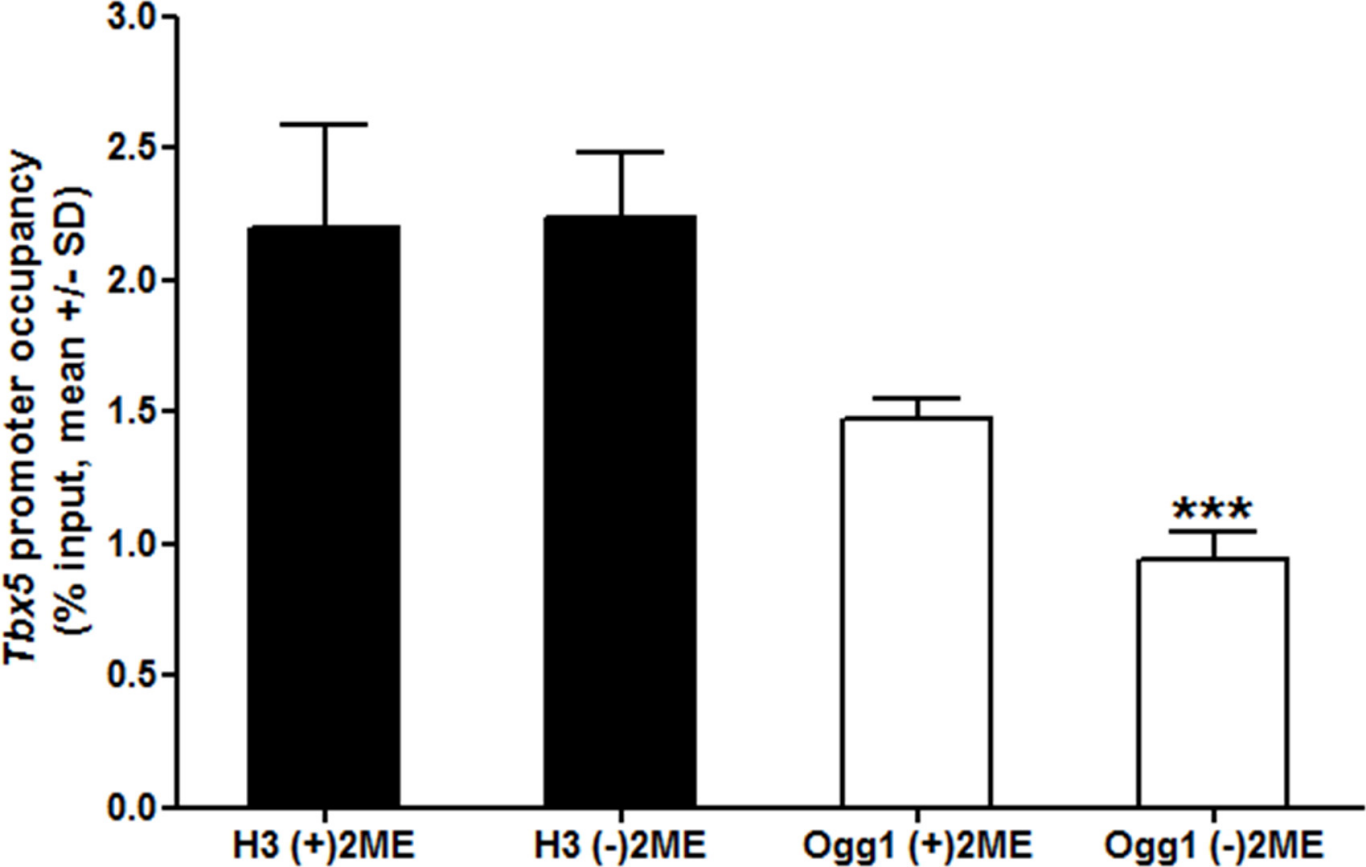
Occupancy of Ogg1 at the *Tbx5* promoter. ChIP-qPCR results showed specific reduced occupancy by Ogg1 (white bars) at the *Tbx5* promoter in oxidative stressed R1 mouse ESCs while occupancy by histone H3 did not change. Mean ± standard deviation (SD), ***p < 0.001, one-sided t-test.

Because *Tbx5* is one of the representative markers of cardiomyocyte lineage differentiation and epigenetic modifications are normally found around its first exon, we detected 8-oxoG in the 5’-proximal region of *Tbx5* exon 1 (bases -1 to +9 from the transcription start site) in ESCs using the newly developed PCR-sequencing assay. PCR products of the target regions of *Tbx5* from ESCs were subcloned, and 16 colonies were subjected to sequencing analysis. Significantly higher numbers of colonies were found to have G∙C to C∙G transversion, but not G∙C to T∙A, at the 7^th^ and 9^th^ base position from transcription start site of *Tbx5* exon 1 in ESCs cultured in (-)2ME than (+)2ME media (*p* < 0.05; Table 2). We also investigated G∙C to C∙G or T∙A transversion surrounding the transcription start sites of *Mef2C*, *Nkx2-5*, and *Gata4* genes. However, in contrast to *Tbx5*, no transversions were observed in these regions (Table 3). ChIP-qPCR assay revealed that the levels of Ogg1 occupancy at the *Tbx 5* promoter decreased by 34% in (-)2ME group compared to the (+)2ME group while no change was detected with histone H3 occupancy at the same promoter region (*p* < 0.001) (Fig 3). Taken together, these results demonstrate that oxidative stress on ESCs induced 8-oxoG formation in the *Tbx5*-specific manner while promoting cardiomyocyte differentiation.

**Table 2:** G∙C to C∙G transversions at exon 1 of the *Tbx5* gene in R1 mouse ESCs subjected to oxidative stress.

Base position from transcription start site^1^	-1	1	3	5	7	9
(+)2ME	2/16	6/16	11/16	11/16	13/16*	13/16*
(-)2ME	1/16	9/16	13/16	14/16	16/16	16/16

^1^G∙C to C∙G transversion were analyzed in a total of 16 colonies.

*p < 0.05, one-sided t-test

**Table 3:** Sequences surrounding the transcription start sites of *Mef2C*, *Nkx2-5*, and *Gata4* genes in R1 mouse ESCs subjected to oxidative stress.

Gene		Sequence
*Mef2C*	Reference	5’-AACCCCCCAGT***T***TGGGCTGCGCTCGCCTCCTCTCCTTTTCTATGAACACCGAAGCTTTGCCCCCTTGATCGTCAAGC-3’
	(+)2ME #1–16	5’-AACCCCCCAGTTTGGGCTGCGCTCGCCTCCTCTCCTTTTCTATGAACACCGAAGCTTTGCCCCCTTGATCGTCAAGC-3’
	(-)2ME #1–16	5’-AACCCCCCAGTTTGGGCTGCGCTCGCCTCCTCTCCTTTTCTATGAACACCGAAGCTTTGCCCCCTTGATCGTCAAGC-3’
*Nkx2-5*	Reference	5’-GTAAACAATATTGTAGTCATAGTCTTGATCCCTGAAAATAACCCCCCTCTTCTGGCTTTCAATCC***A***TCCTCACCAGCC-3’
	(+)2ME #1–16	5’-GTAAACAATATTGTAGTCATAGTCTTGATCCCTGAAAATAACCCCCCTCTTCTGGCTTTCAATCCATCCTCACCAGCC-3’
	(-)2ME #1–16	5’-GTAAACAATATTGTAGTCATAGTCTTGATCCCTGAAAATAACCCCCCTCTTCTGGCTTTCAATCCATCCTCACCAGCC-3’
*Gata4*	Reference	5’-TCCACACGTACCAAGGAGAAAAAGCCAGCCACCAGCCACGGG***A***CTCGCTGAATTAATGAATCACTTTTCTTATCT-3’
	(+)2ME #1–16	5’-TCCACACGTACCAAGGAGAAAAAGCCAGCCACCAGCCACGGGACTCGCTGAATTAATGAATCACTTTTCTTATCT-3’
	(-)2ME #1–16	5’-TCCACACGTACCAAGGAGAAAAAGCCAGCCACCAGCCACGGGACTCGCTGAATTAATGAATCACTTTTCTTATCT-3’

***Bold italic*** bases indicate putative transcription start sites (*Mef2C*, *Nkx2-5*, *Gata4*)

**Fig 4:**
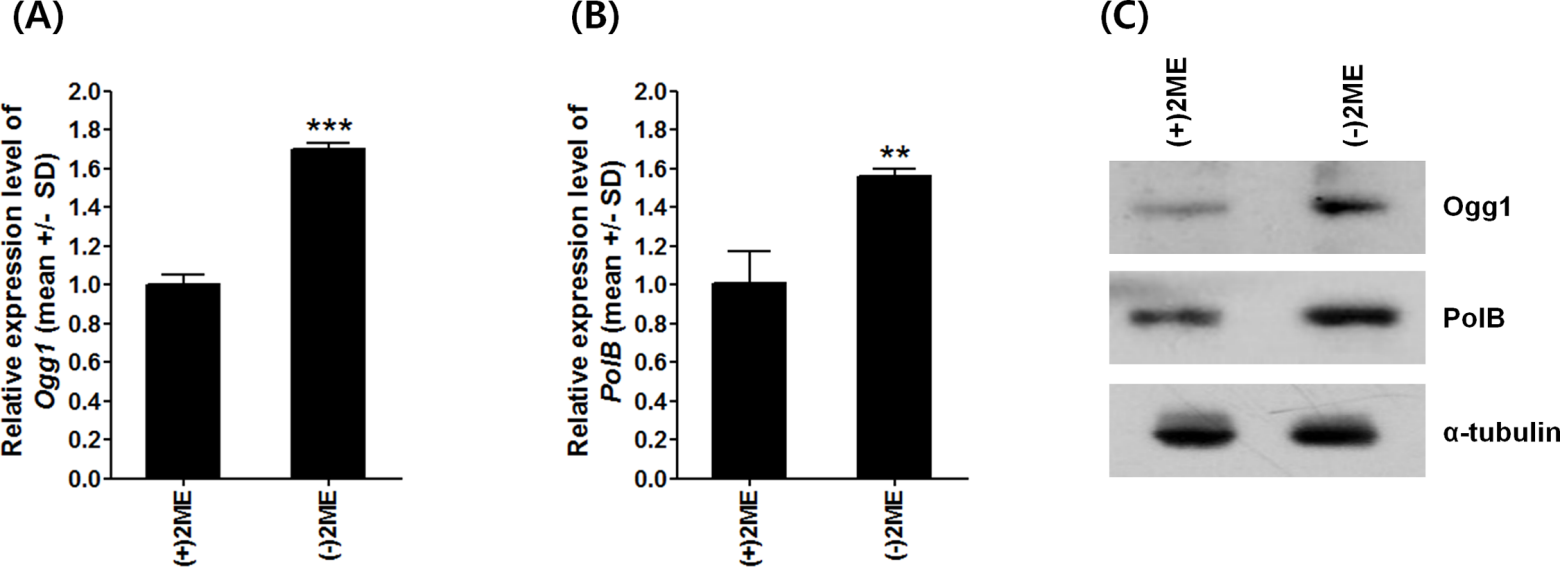
Differential expression levels of 8-oxoG repair genes *Ogg1* and *PolB* in ESCs induced by oxidative stress. Relative mRNA levels of (A) *Ogg1* mRNA and (B) *PolB*. Gapdh was used as an internal control. Mean ± SD, **p < 0.01, ***p < 0.001, one-sided t-test. (C) Levels of Ogg1 and PolB proteins by Western blotting. α-tubulin was used as an internal control.

The 8-oxoG formation was also accompanied by up-regulation of 8-oxoG recognizing genes such as *Ogg1* and *PolB*. qPCR revealed that the levels of mRNA for *Ogg1* in ESCs cultured in (-)2ME medium was 1.70 ± 0.03 times higher than those in the (+)2ME group (*p* < 0.01) (Fig 4A). Similarly, the mRNA levels of *PolB* significantly increased in ESCs cultured in (-)2ME medium when compared to those in (+)2ME medium (Fig 4B). These increased mRNA levels were accompanied by increases in the protein levels of both *Ogg1* and *PolB*. Specifically, the levels of Ogg1 and PolB proteins in ESCs increased 2.2- and 1.3-fold, respectively, when media were changed from (-)2ME to (+)2ME (Fig 4C).

## Discussion

Despite the availability of several methods for the detection of 8-oxoG in DNA, they are either qualitative or indirect. Quantifying 8-oxoG lesions in specific genes in the genome has therefore been challenging. Here, we established an assay using the intrinsic property of 8-oxoG that allows it to pair with adenine rather than cytosine through Hoogsteen base pairing. It thus enables us to detect 8-oxoG lesions in DNA at specific bases by strategically-designed PCR reaction. Using plasmid DNA containing an 8-oxoG lesion, we could detect the default G∙C to T∙A transversions caused by 8-oxoG in the PCR products.

In *in vivo* analysis, we unexpectedly detected G∙C to C∙G transversion, not just G∙C to T∙A, in loci of *Tbx5* exon 1 in ESCs under oxidative stress. A simple explanation of the different transversion findings in this study might be the formation of imidazolone from 8-oxoG. Imidazolone is made by further oxidation of 8-oxoG and such a change causes incorporation of a G to the opposite strand during replication. Consequently, imidazolone-G base pairs result in G∙C to C∙G transversion [32–34]. Compared to G∙C to T∙A transversion, the frequency of G∙C to C∙G transversion is considerably higher under oxidative stress [35, 36]. Furthermore, complex redox reaction as well as antioxidant defense systems likely operate in ESCs, which further drive oxidation of 8-oxoG to imidazolone. Compared to this *in vivo* system of ESCs resisting oxidative stress, there are no artificial or intrinsic components in the simple PCR assays to catalyze additional oxidation that occurred in ESCs under stress. In support of this logic, no additional oxidative damage has been reported on naked DNA during PCR. Hence, only G∙C to T∙A transversion was observed from the bared plasmid DNA while mainly G∙C to C∙G transversions were seen in oxidative-stress ESCs. Although PCR can cause errors particularly at mono- and dinucleotide repeats [37], the combined observations that we only observed G∙C to C∙G in oxidative-stressed ESCs but not in amplification of the plasmid DNA by PCR, the G∙C to C∙G transversion could only be resulted from further oxidation of 8-oxoG to imidazolone in ESCs even we did not evaluate the formation of imidazolone in DNA of these cells. The results here also demonstrated that G∙C to C∙G transversion during cellular replication could be used as an alternative marker of 8-oxoG lesion.

We evaluated 8-oxoG modifications surrounding the transcription start sites of *Mef2C*, *Nkx2-5* and *Gata4*. Unfortunately, we could not find any 8-oxoG modification-related G:T or G:C conversion in any of them. It is likely that different regulation mechanisms were exerted on different genes. For example, it has been reported that *Nkx2-5* is regulated by histone modification [38] and DNA methylation [39]. *Mef2C* is upregulated by HDAC inhibition, implying that histone modification is a regulator of its activity [40]. *Gata4* expression is also regulated by histone modification [41]. Therefore, among genes that were studied, guanine oxidation-mediated gene expression is limited to *Tbx5* during myocardial specification of mouse ESCs. Our ChIP-qPCR data support the conclusion of *Tbx5*-specific 8-oxoG modification by revealing reduced occupancy of Ogg1 at *Tbx5* promoter in the oxidative stress-induced ESCs. These observations are consistent with the previous findings that genomic integrity is significantly reduced by oxidative stress, and Ogg1-mediated single-stranded break at the apurinic site would decrease the amplifiable amount of DNA [42, 43].

Apart from measuring 8-oxoG formation by directly detecting base pair transversion, base excision repair for 8-oxoG lesions also provides an indirect measure for 8-oxoGs formation in DNA. For instance, 8-oxoGs are specifically recognized and removed by Ogg1. After removal of 8-oxoG and its sugar backbone, PolB is recruited for DNA replication generating single nucleotide gap, and gap sealing is followed to complete the base excision repair [44]. Perillo and colleagues reported that an estrogen-induced burst of nuclear 8-oxoGs facilitates Ogg1 recruitment to the promoter and estrogen response elements genes, such as *Bcl2* [17], indicating that analyzing the expression levels of 8-oxoG repair enzymes could be used as an indicator for the generation of 8-oxoG lesions. Consistent with this finding, we observed that the mRNA levels for *Ogg1* and *PolB* were significantly increased in oxidative-stressed ESCs. Accompanied with the detection of 8-oxoG by PCR-sequencing assay, these result support that oxidative stress might induce 8-oxoG in DNA of ESCs, and enzymes participating in 8-oxoG recognition and repair are effective in qualifying 8-oxoG lesions in DNA.

In the present study, we introduced oxidative stress to ESCs by altering culture media composition. 2ME, a reducing agent, is a common ingredient of ESC culture medium. It has been reported that a burst of oxidative stress is induced in mouse ESCs by simple removal of 2ME from the culture medium [45], which activates apoptosis in ESCs featured with nucleus fragmentation and DNA degradation. Although oxidative stress is involved in signal transduction rather than simply a cue for cell death, apoptosis has been regarded as a hall mark for oxidative stress in ESCs [45]. Therefore, we confirmed that oxidative stress could be induced in ESCs by simple removal of 2ME from culture media, resulting in significant lower proliferation. In addition, we demonstrated that 8-oxoG induced by oxidative stress could be transmitted to progeny cells to influence cardiomyocyte specification after onset of random differentiation. The incidence of beating EBs derived from physiological glucose medium without 2ME was significantly higher than the medium with 2ME. It was consistent with the previous reports that ROS stimulates ESCs-derived cardiomyogenesis. For example, low levels of H_2_O_2_ stimulated cardiomyogenesis of ESCs and induced proliferations of cardiomyocytes derived from ESCs as investigated by nuclear translocation of cyclin D1, down-regulation of p27 (Kip1), and retinoblastoma phosphorylation, and increased of Ki-67 expression [46]. Furthermore, intracellular ROS shows a trend to increase during the process of ESC differentiation and has an essential role in cardiomyocyte differentiation of ESCs [28, 47–49]. These findings imply that ROS can act as signals for cardiomyocyte differentiation and growth. However, in this study we eliminated oxidative stress during EB formation by including antioxidant in the differentiation medium, any sustaining effects of oxidative stress could not be resulted from short-lived signal molecules. Rather, changes in EB formation and differentiation should be resulted from more permanent changes such as oxidative guanine modification. Together, our results demonstrate that oxidative guanine persisted in progenies of treated ESCs and promoted differentiation of ESCs to cardiomyocytes by epigenetic mechanisms.

In conclusion, we developed alternative methods to investigate oxidative guanine qualitatively and quantitatively by using base excision repair-related gene expression analysis and PCR-sequencing assay. Additionally, oxidative stress-induced 8-oxoG formation might contribute to the favored differentiation of ESCs into cardiomyocytes. These understandings of epigenetics will shed lights to mammalian development and regenerative medicine.
